# Necrotizing Fasciitis: A Life-Threatening Infection Due to Clostridium Species

**DOI:** 10.7759/cureus.22315

**Published:** 2022-02-17

**Authors:** Sharon Hechter, Vraj Patel, Veera Jayasree Latha Bommu, Priya Patel, Xue Ao, Dina Alnabwani, Pramil Cheriyath

**Affiliations:** 1 Internal Medicine, Hackensack Meridian Ocean Medical Center, Brick, USA

**Keywords:** gas gangrene, clostridium septicum, nsti, necrotizing soft tissue infection, nf, necrotizing fasciitis

## Abstract

Necrotizing fasciitis (NF), soft tissue infections, are rare but rapidly progressive and life-threatening infections with high morbidity and mortality rates. Early detection and intervention by physicians are paramount in mortality prevention. We present a case report of a 77-year-old female who presented with extensive NF due to a Clostridium septicum infection.

## Introduction

The bacterial infection, necrotizing soft tissue infection (NSTI) is characterized by fast and widespread necrosis from the epidermis to the deep muscles. The global incidence of NSTI ranges from 0.3 to 5.0 cases per 100,000 individuals [1]. Based on the existence of polymicrobial or monomicrobial infection, necrotizing fasciitis (NF) is categorized into categories I and II [1]. NF can affect any area of the body; however, they are most usually found in the extremities, particularly the lower limbs [2]. It appears to favor males, with a male to female ratio of 3:1; this ratio is mostly linked to an increased incidence of Fournier's gangrene in males. The condition affects people of all ages, however, it is more common in middle-aged and elderly people (over 50 years old), especially diabetic patients [3].

The majority of NSTIs are caused by a combination of aerobic and anaerobic bacteria that work together to generate a fulminant illness. Monomicrobial necrotizing infections include myonecrosis (gas gangrene) caused by Clostridium infection and NF caused by group A Streptococcus [4]. Although Clostridium septicum is responsible for a tiny percentage of clostridial infections, it is associated with a high risk of morbidity and mortality [5].

## Case presentation

A 77-year-old female presented to the Emergency Department (ED) with severe pain and left leg swelling for the past three days. She was unable to move the left leg. Her pain started gradually three days back and became worse, over the left lower quadrant (LLQ) of abdomen, left leg, with no radiation. The patient denied any numbness or tingling in the left leg or other extremities, nausea, vomiting, or diarrhea. On clinical examination, she was alert and oriented, tenderness was elicited over LLQ with no pulsatile mass. She had extensive discoloration and edema of the left thigh, gluteal area, and leg extending down to her left knee. She was able to contract left quadriceps and hamstrings but was unable to lift the left leg secondary to abdominal pain. Reviews of other systems were unremarkable.

Her past medical history includes a history of stage IV colorectal cancer with liver metastasis in remission, radiation to the perineum, pelvic area, partial hepatectomy, or segmentectomy, breast reduction surgery. She was allergic to percocet (oxycodone-acetaminophen). She reported that she has quit smoking, never used smokeless tobacco but was using alcohol. Her labs are demonstrated in Table 1.

**Table 1:** Patient’s laboratory findings and reference range

Labs	Patient’s value	Reference Range
White blood cells (WBC)	3.3 10^3^/µL	4.5 - 11.0 10^3^/µL
Fasting serum glucose	256 mg/dL	70 - 99 mg/dL
Blood urea nitrogen (BUN)	29 mg/dL	5 - 25 mg/dL
Serum creatinine	1.90 mg/dL	0.44 - 1.00 mg/dL
Alanine aminotransferase (ALT)	145 U/L	10 - 60 U/L
Aspartate aminotransferase (AST)	295 U/L	10 - 42 U/L
Total bilirubin	1.6 mg/dL	0.0 - 1.3 mg/dL
Sodium	133 mmol/L	136 - 145 mmol/L
Potassium	3.5 mmol/L	3.5 - 5.2 mmol/L
Chloride	92 mmol/L	96 - 110 mmol/L
Carbon dioxide	14 mmol/L	24 - 31 mmol/L
International normalized ratio (INR)	1.43	0.88 - 1.15
Prothrombin time (PT)	16.1 sec	9.8 - 12.9 sec

Anion gap was elevated at 27.0 (5-13 mmol/L), estimated glomerular filtration rate (EGFR) non-African-American of 26 (>60 mL/min/1.73 m^2^), and troponin 0.11 (<0.04 ng/mL). Arterial blood gas (ABG) showed low pH of 7.338 (7.350-7.450), pCO_2_ of 21.5 (35.0-45.0 mmHg), pO_2_ of 117.5 (75.0-100.0 mmHg), HCO_3_ 11.3 (22.0-28.0 mmol/L), elevated serum lactic acid of 10.0 (0.5-2.0 mmol/L), and creatinine kinase (CK) of 12,932 U/L (22 to 198 U/L). Electrocardiogram (EKG) showed a heart rate of 124 beats per minute, sinus tachycardia, with non-specific ST-T-wave changes. Intravenous fluids, vancomycin, piperacillin-tazobactam, morphine, and ondansetron were administered. 

After a few hours, she became unresponsive and apneic due to which she was intubated in ER. She was unstable on Levophed (Norepinephrine). On repeat examination, the upper abdomen was soft, non-tender, non distended, no rebound, no guarding, no crepitance. Edema, swelling from lower pelvis, perineum extending down to the left quadriceps area and left gluteal area, there is reddish-blue discoloration with bullae, tense, not crepitant, extended all the way to the left inferior gluteal fold towards the perineum. She had the spontaneous movement of her left leg. Edema was noted extending all the way down to the knee.

**Figure 1:**
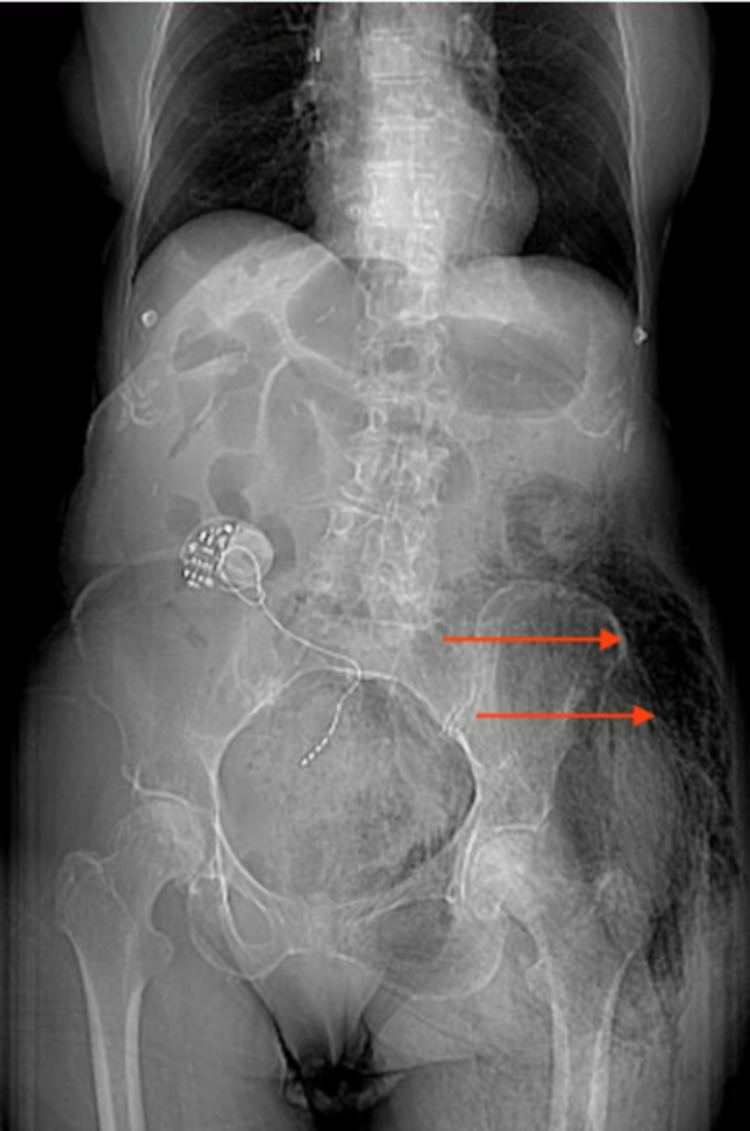
Computerized tomography (CT) abdomen and pelvis with contrast anteroposterior view showing free air in left gluteal and hip regions (red arrows)

**Figure 2:**
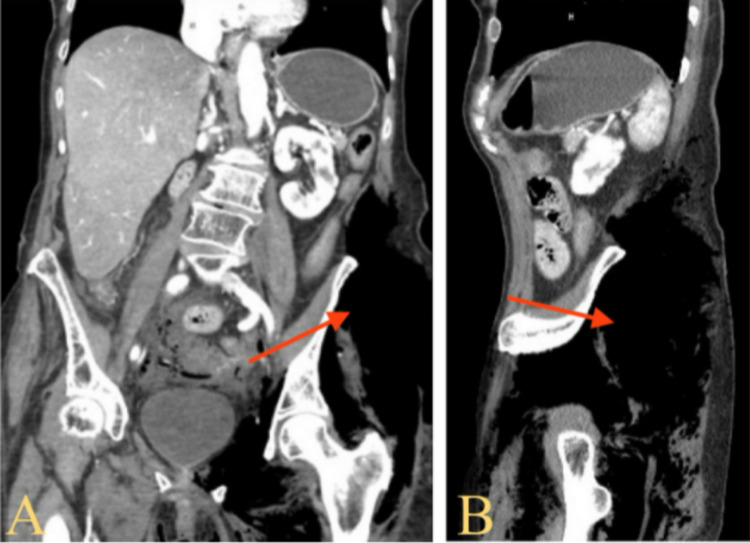
Computerized tomography (CT) abdomen and pelvis with contrast anteroposterior (A) and lateral (B) view showing extensive surgical emphysematous changes within the left-sided gluteal muscles along the posterolateral aspect of the left hip joint and anterolateral aspects of the left thigh (red arrows), extending to the posterior lower spinal region and anteriorly along left-sided pelvic wall with extension into the abdominal cavity with pneumo-retroperitoneum

Computerized tomography (CT) abdomen and pelvis with contrast revealed extensive surgical emphysematous changes within the left-sided gluteal muscles along the posterolateral aspect of the left hip joint and anterolateral aspects of left thigh, extending to posterior lower spinal region and anteriorly along left-sided pelvic wall with extension into abdominal cavity with pneumo-retroperitoneum (Figures 1, 2A, 2B).

**Figure 3:**
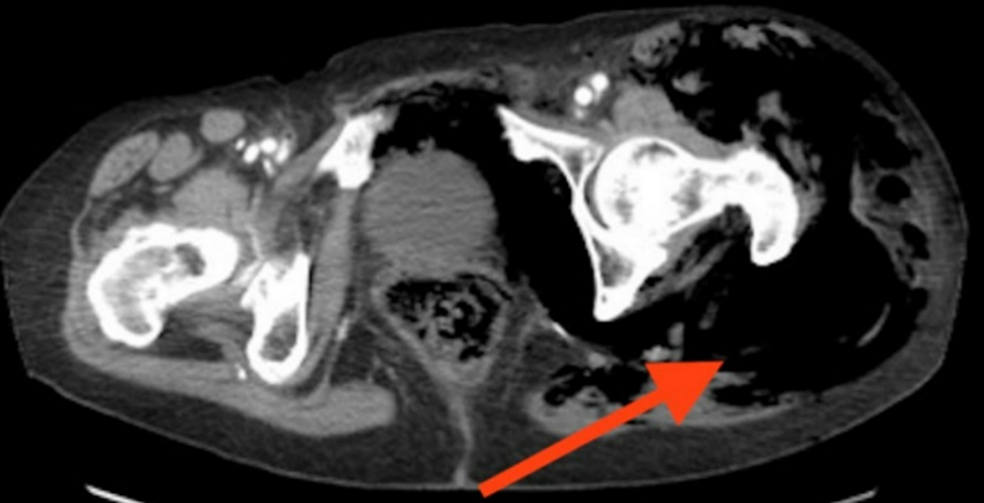
Computerized tomography (CT) pelvis transverse view showing free air in the left gluteal area (red arrow)

CT shows extensive free air extending from the pelvic area, lower rectal area into the entire left gluteal area and anteriorly (Figure 3). There was almost no noticeable muscle. NF should be considered, a minimal amount of ascites, degenerative lumbar spondylosis with changes of facet joint arthropathy. Levoscoliotic deformity of the lumbar spine. Diffuse osteopenia, diffuse atherosclerotic wall calcifications involving the aorta, its branches, and its bifurcation. Duplex Doppler scan of lower extremity did not identify any evidence of arterial vascular compromise.

She was diagnosed with NF with overwhelming sepsis, left leg compartment syndrome, respiratory arrest. Prompt surgical intervention was initiated and the patient was taken to the operating room for life-saving measures. Intraoperatively, the patient had extensive liquefied necrolytic changes of the lateral gluteal vastus lateralis portion of the lateral aspect of the rectus femoris requiring aggressive debridement. There was no abscess. There was extensive gas when we performed a fasciotomy. She underwent aggressive three-compartment fasciotomies, debridement of liquefied necrotic fascia, and was brought into the intensive care unit in critical condition. After the surgery, she had a much better range of motion in the left leg. She was taken to the recovery room in critical condition. A swab from her left thigh and blood cultures came out positive for Clostridium septicum. The patient was orally intubated, unresponsive to verbal and tactile stimuli. The patient's family chose comfort care measures for the patient so she was palliatively extubated and expired thereafter.

## Discussion

NSTIs are a relatively uncommon clinical condition, with an annual global incidence of about 0.4 per 100,000 [6]. Necrotizing infections can develop after major traumatic injuries, as well as minor skin or mucosa breaches (e.g., tears, abrasions, lacerations, or insect bites), varicella infection, nonpenetrating soft-tissue injuries (e.g., muscle strain or contusion), or routine obstetrical and gynecologic procedures; they can also develop in postsurgical and immunocompromised patients [7].

Diabetes mellitus (DM) is the most common comorbidity in patients with NF with a frequency of 40%-60%. Cirrhosis of the liver, chronic heart failure, obesity, alcohol misuse, immunodeficiency, systemic lupus erythematosus, Addison's disease, pre-existing hypertension, and peripheral vascular disease are all other common comorbidities. Although debatable, advanced age is another risk factor for increased incidence and death [8]. Age is a powerful, independent predictor of mortality, according to large population-based research [9]. According to Rea and Wyrick's research, patients over the age of 50 have a 67% mortality rate, while those under the age of 50 had a 4% mortality rate [10].

NF is categorized into two types based on the cause, which is polymicrobial or monomicrobial. Type I NF is a polymicrobial illness that includes both aerobic and anaerobic bacteria. It is most common in the elderly or those who have underlying ailments. Diabetic or decubitus ulcers, hemorrhoids, rectal fissures, episiotomies, colonic or urologic surgery, and gynecologic procedures are all predisposing factors [7]. Type II NF is a monomicrobial illness. The most frequent gram-positive pathogen is group A streptococcus, followed by methicillin-resistant Staphylococcus aureus (MRSA). Type II infections, unlike type I infections, can affect people of any age and without any underlying sickness. Aeromonas hydrophila and Vibrio vulnificus are two other pathogens [7].

Some researchers believe that infections caused by these microorganisms, as well as perhaps clostridial species, should be categorized as NF type III. Monomicrobial infections caused by Clostridium species or gram-negative bacteria are classified as Type III. External traumas (deep wounds or crush injuries producing local devascularization) or surgical wounds can produce Clostridium species, which are anaerobic bacteria (intestinal and obstetric). Clostridium infections are becoming more common among drug addicts, with Clostridium perfringens being the most common of the C. species [8]. Clostridial infections, most often Clostridium perfringens, can emit neurotoxic exotoxins and phytotoxins, which can cause gas gangrene, NSTI, and death [5].

Infection tends to migrate quickly across the fascia planes, causing progressive fascia degradation at a pace of two to three cm per hour. Its rapid clinical progress is linked to polymicrobial infection and synergy, which generally co-exists in the lower or higher limbs, the perineum, and genital area (Fournier's gangrene), and the abdominal wall [8]. The majority of cases present anaerobic bacteria that proliferate in a hypoxic environment and produce gas, which accumulates in the soft tissue spaces, giving the characteristic image of gas gangrene on plain x-rays and CT scans [7]. Soft-tissue edema is seen in most cases, followed by erythema, severe pain, soreness, fever, and skin bullae or necrosis are all common symptoms of NF [7].

Prompt, aggressive surgical debridement of all devitalized tissue and high-dose antibiotic therapy in intensive care units (ICU) appear to provide patients with the best chance of survival [11]. Clindamycin and penicillin, which cover the Clostridium species, should be used to treat category III NF. The primary aspects of surgical treatment include surgical debridement, necrosectomy, and fasciotomy as early as possible. Untreated NF can result in life-threatening complications such as systemic inflammatory response syndrome (SIRS) or multi-organ dysfunction syndrome (MODS) with a mortality rate of 70% [8].

## Conclusions

NF is a life-threatening infection that can be polymicrobial or monomicrobial. Clostridium species such as clostridium septicum are one of the rare causes of NF. Our patient was infected with clostridium septicum that was complicated with septicemia and succumbed to death. Early aggressive surgical debridement along with intravenous antibiotics can improve chances of survival.

## References

[1] Cui Z, Lu S, Bai Y (2021). Necrotizing soft tissue infection: clinical characteristics, diagnosis, and management of 32 cases in Beijing. J Int Med Res.

[2] de Prost N, Lipman J, Mimoz O (2017). Therapeutic targets in necrotizing soft tissue infections. Intensive Care Med.

[3] Levine EG, Manders SM (2005). Life-threatening necrotizing fasciitis. Clin Dermatol.

[4] Urschel JD (1999). Necrotizing soft tissue infections. Postgrad Med J.

[5] McDonald RE, Moola S (2012). Clostridium septicum infection in a young pregnant patient. BMJ Case Rep.

[6] Leiblein M, Wagner N, Adam EH, Frank J, Marzi I, Nau C (2020). Clostridial gas gangrene - A rare but deadly infection: case series and comparison to other necrotizing soft tissue infections. Orthop Surg.

[7] Stevens DL, Bryant AE (2017). Necrotizing soft-tissue infections. N Engl J Med.

[8] Misiakos EP, Bagias G, Patapis P, Sotiropoulos D, Kanavidis P, Machairas A (2014). Current concepts in the management of necrotizing fasciitis. Front Surg.

[9] Sorensen MD, Krieger JN, Rivara FP, Klein MB, Wessells H (2009). Fournier's gangrene: management and mortality predictors in a population based study. J Urol.

[10] William JR, Walter JW (1970). Necrotizing fasciitis. Ann Surgery.

[11] Mittermair RP, Schobersberger W, Hasibeder W, Allerberger F, Peer R, Bonatti H (2002). Necrotizing fasciitis with Clostridium perfringens after laparoscopic cholecystectomy. Surg Endosc.

